# Identification of novel mutations and phenotype in the steroid resistant nephrotic syndrome gene *NUP93*: a case report

**DOI:** 10.1186/s12882-019-1458-z

**Published:** 2019-07-17

**Authors:** Ibrahim Sandokji, Jonathan Marquez, Weizhen Ji, Cynthia A. Zerillo, Monica Konstantino, Saquib A. Lakhani, Mustafa K. Khokha, Jillian K. Warejko

**Affiliations:** 1 Department of Pediatrics, Section of Nephrology, Yale University School of Medicine, 333 Cedar St., PO Box 208064, New Haven, CT 06520-8064 USA; 2 Pediatric Genomics Discovery Program, Department of Pediatrics and Genetics, Yale University School of Medicine, New Haven, CT USA

**Keywords:** Steroid-resistant nephrotic syndrome, Focal segmental glomerulosclerosis, Whole exome sequencing, Genetics, Inherited diseases

## Abstract

**Background:**

Monogenic mutations may be a significant cause of steroid-resistant nephrotic syndrome. *NUP93* is a gene previously reported to cause isolated steroid-resistant nephrotic syndrome.

**Case presentation:**

Here we describe a case of recessive, syndromic, steroid-resistant nephrotic syndrome caused by *NUP93* mutation.

**Conclusions:**

*NUP93* may convey a phenotype that has not only SRNS, but also other syndromic features.

## Background

Steroid therapy is a mainstay of treatment for nephrotic syndrome. However, in 15–20%, there is no response to steroid therapy [1], increasing the risk of developing end-stage renal disease (ESRD) and requiring renal replacement therapies during the first two decades of life [2]. In 11–30% of steroid-resistant nephrotic syndrome (SRNS), a known gene mutation can be detected [3]. *NUP93* is a widely expressed gene that encodes a highly conserved nuclear pore protein. Knockdown of *NUP93* leads to inhibition of podocytes proliferation by impairing SMAD signaling resulting in focal segmental glomerulosclerosis (FSGS). Mutations of *NUP93* have been shown to cause non-syndromic autosomal recessive FSGS, that can progress to ESRD within ten years [4, 5]. Here we describe a case of novel *NUP93* mutations in a child with a syndromic SRNS phenotype.

## Case presentation

**Fig. 1:**
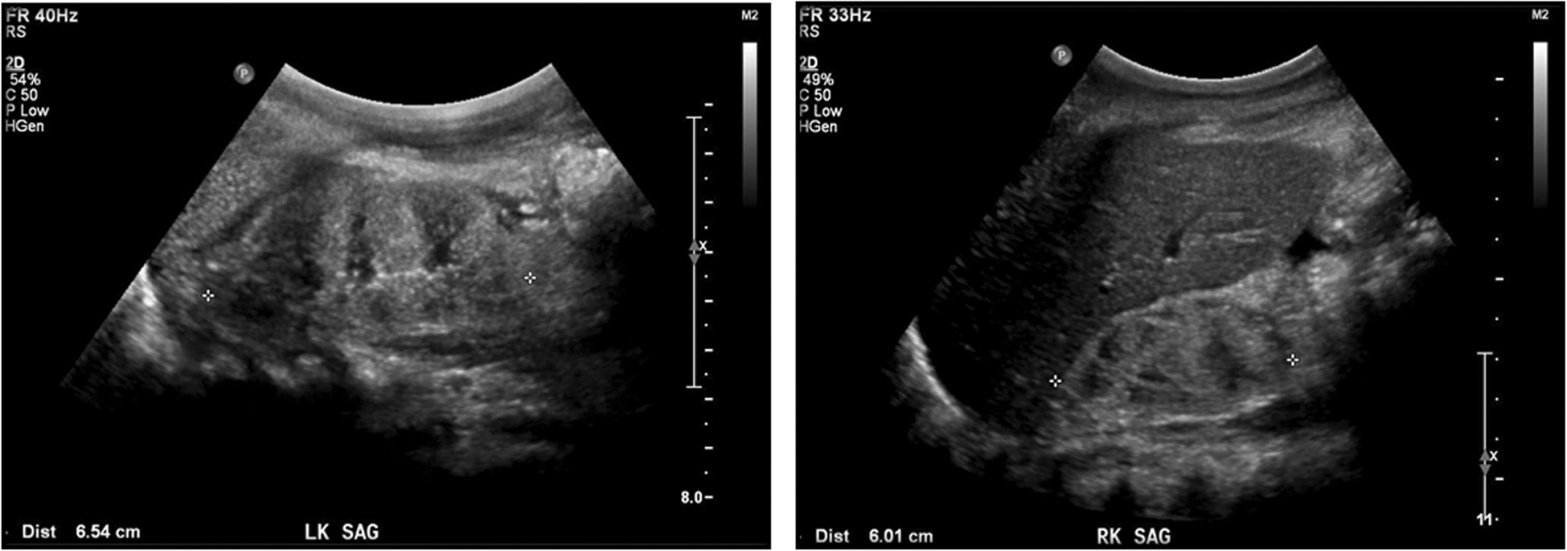
Renal ultrasound at presentation. The patient’s kidneys were notable for small size, diffuse echogenicity and loss of corticomedullary differentiation. Average kidney size for 5-year-old children is 8 cm, however she was small for age, and for a 90 cm tall child the average kidney size is 7.1 cm with 95% prediction limits of 5.8 to 8.3 cm. The ultrasound was performed at the time she was anuric, hence the renal pelvis appears to be collapsed

A 5-year-old nonconsanguineous girl of African American and Hispanic origin presented with nephrotic syndrome, including nephrotic-range proteinuria (UPC of > 29 mg/mg), edema, and hypoalbuminemia. Her initial serum creatinine was 654 μmol/L. Other pertinent laboratory evaluation at time of presentation included albumin of 19 g/L, BUN of 38 mmol/L, potassium of 6 mmol/L, bicarbonate of 12 mmol/L, calcium of 1.7 mmol/L, phosphorus of 2.5 mmol/L, and parathyroid hormone of 396 ng/L. She was oligoanuric and hemodialysis was initiated. An ultrasound of her kidneys showed diffuse echogenicity and loss of corticomedullary differentiation (Fig. 1). Her history was significant for developmental delay and short stature. Her proteinuria presented in the setting of a previous respiratory illness but was not investigated. She has a normal-looking face and without dysmorphic features which was confirmed by the hospital’s geneticist. An ophthalmological examination did not show cataract or retinal changes. She has normal looking ears and exhibited normal hearing. She was normocephalic and did not have an exam consistent with GAMOS and no uro-genital anomalies were identified. She had normal birth history, and her family history was not significant for renal, cardiac or neurological development problems.

**Fig. 2:**
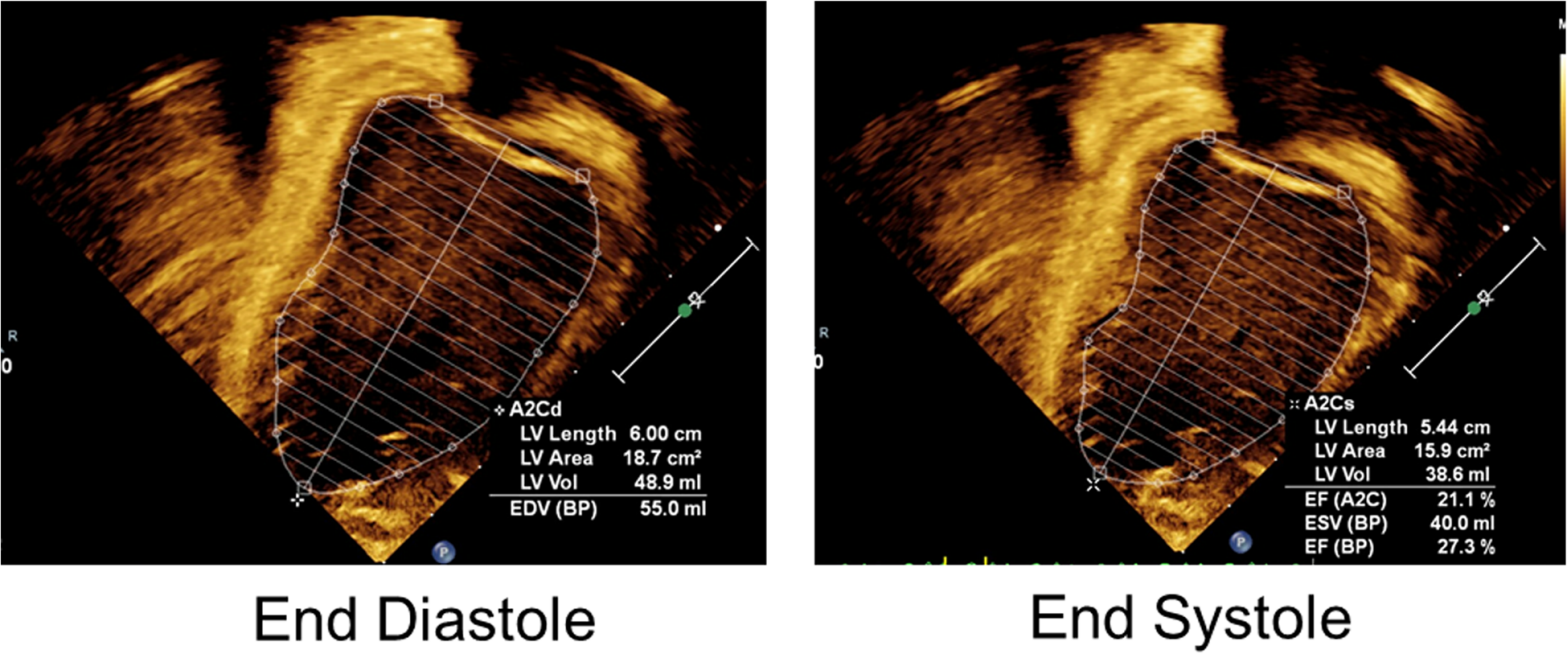
Patient echocardiogram. An apical, left two-chamber view shows moderately dilated left ventricle, mild concentric left ventricular hypertrophy and severely reduced systolic function. Left ventricular end diastolic diameter Z score 5.59 by M-mode and end-diastolic volume by 5/6 area length method is Z score 4.98. left ventricular dp/dt of 1398. There is also a LV diastolic dysfunction with fusion of E and A waves of inflow doppler pattern. There is moderate to severe mitral valve regurgitation and mild to moderate tricuspid regurgitation. The aortic and pulmonary valves are trivially regurgitant. There is no pericardial effusion

In addition to her kidney involvement, she had developmental delays with autistic features; including delays in expressive language, fine motor, social communication and repetitive hand movements. She had expressive, receptive, and pragmatic language difficulties with a low score in the auditory comprehension subtest of the Preschool Language Scales. Additionally, while awaiting renal transplant, she had two episodes of heart failure requiring inotropic support after having adequate dialysis for more than a month. She had severely elevated B-type natriuretic peptide (BNP) levels (> 70,000 pg/mL) and her echocardiogram showed systolic and diastolic dysfunction (ejection fraction as low as 35%) and dilated cardiomyopathy features (Fig. 2). After receiving aggressive nutritional support and blood pressure management, her cardiac function improved with ejection fractions range in 40–50%. She received a living related kidney transplant without recurrence of cardiac symptoms, and normal cardiac structures on echocardiograms with ejection fractions > 60%. Given that we know NUP93 does localize to cilia in *Xenopus* during cardiac development, we cannot exclude there may have been a contribution of the patient’s mutations to this phenotype. After her kidney transplant, she did not have recurrence of her nephrotic syndrome, but had a brief period of proteinuria (maximum UPC of 3.6) that was monitored closely and resolved within one week.

**Fig. 3:**
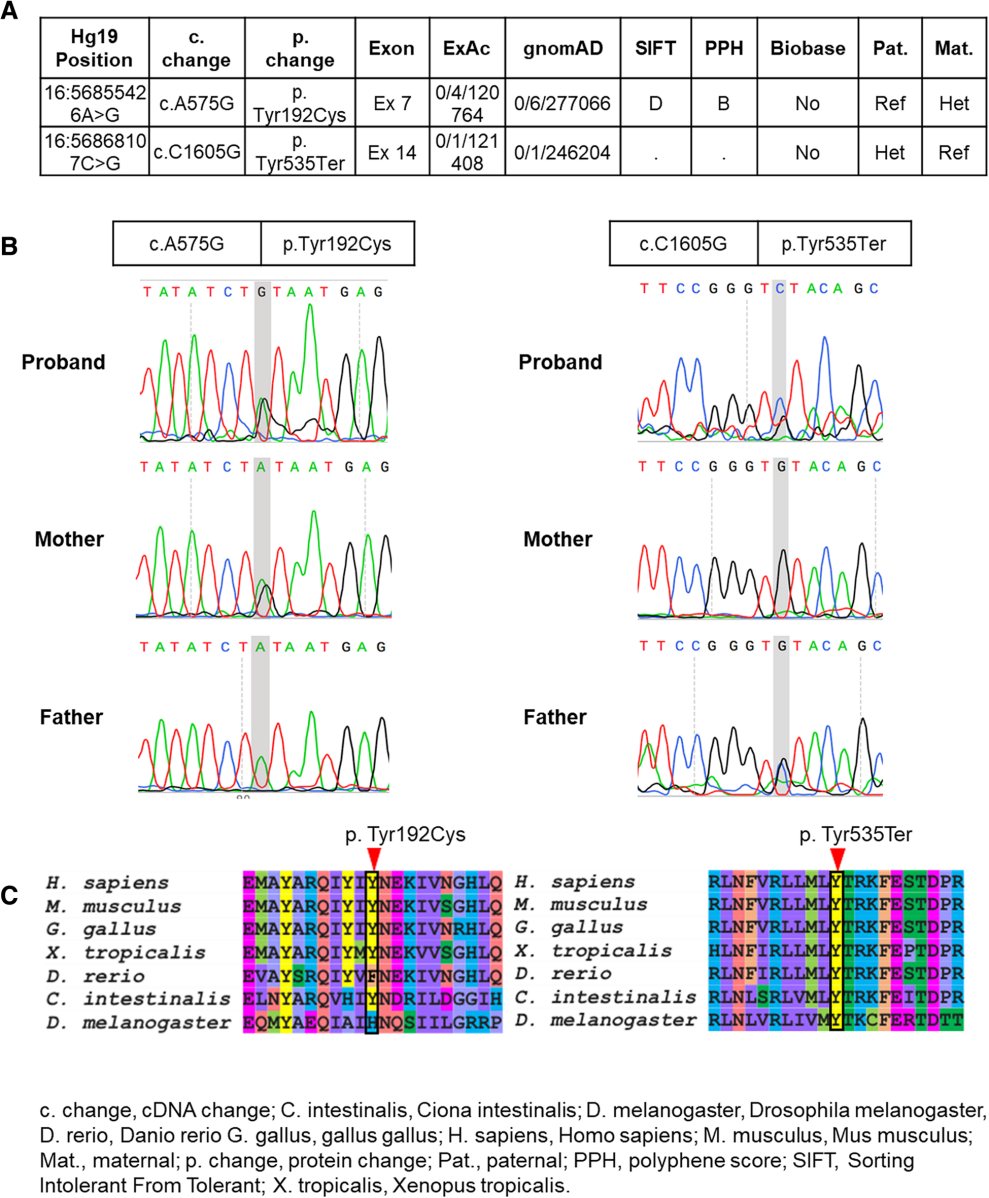
Genotype and conservation of proband’s mutations in *NUP93*: **a**. Table with Hg19 position, c, change; ExAC, Exome Aggregation Consortium; gnomAD, Genome Aggregation Database; p. change, exon number, mean allele frequency, pathogenicity prediction scores, and segregation for the two mutant alleles in our proband. **b**. Sanger tracing for proband, mother and father for each allele. **c**. Conservation through phylogeny for the two mutated alleles of *NUP93*

Given her presentation of likely hereditary nephrotic syndrome, we sent clinical whole exome sequencing (WES). WES demonstrated a compound heterozygous mutations in *NUP93*; a maternal missense variant (chr16:56855426 A > G) c.A575G, p. Tyr192Cys and a paternal nonsense variant (chr16:56868107 C > G) c.C1605G, p. Tyr535Ter (Fig. 3). Both variants are extremely rare, only 6 alleles of p.Tyr192Cys and 1 allele of p.Tyr535Ter have been reported previously in a large population database (gnomAD) with over 246,000 chromosomes, with a higher frequency of p.Tyr535Ter in African population (1 in 16,256). These allele frequencies are < 0.1%, which we have previously used as a cut off for filtering potentially pathogenic alleles [6]. The locations of the two variants are in the α helical domain of the NUP93 protein, as are some of the previously reported pathogenic mutations in Braun, et al. [5]. The tyrosine at position 192 is conserved through phylogeny, and the missense variant Tyr192Cys had high impact prediction scores for deleteriousness by CADD or SIFT. The nonsense variant p. Tyr535Ter likely results in defective protein structure either through truncation or nonsense-mediated mRNA decay. Additional analysis of her WES did not identify mutations related to cardiomyopathy.

## Discussion and conclusions

We identified novel compound heterozygous mutations of *NUP93* gene using WES in a patient with nephrotic syndrome progressing to ESRD in the first decade of life. Fourteen patients were reported to have *NUP93* mutations associated with SRNS [4–6]. Nup93 variants were first reported in 9 families with isolated SRNS [5]. Rapid progression into ESRD was also observed in a subsequent case series of genetic SRNS due to *NUP93* mutations [3, 4, 6].

As part of the nuclear pore complex, NUP93 protein functions in transport between the cytoplasm and nucleus and was shown to have a significant role in the SMAD signaling pathway in *Drosophila* [7]. Braun et al. studied the role of NUP93 in immortalized human podocytes, and demonstrated a critical role for NUP93 in bone morphogenetic protein 7 (BMP7)-dependent SMAD signaling, a novel pathway for SRNS [5]. This interaction supports the role of BMP7 in renal response to injuries and development of chronic kidney changes. The defect of SMAD signaling was shown in all *NUP93* knock out cells and resulted in reduced podocyte proliferation. In addition, Nup93 is essential for cilia and cardiac development in *Xenopus* and may play a role at the cilium base that is independent of its role in the nuclear pore [8]. Loss of cilia can cause a cardiomyopathy in mice suggesting a potential pathogenic mechanism for the cardiomyopathy in our patient [9]. Another nucleoporin mutation, NUP107, was reported in one child with dilated cardiomyopathy indicating the potential role of ciliopathies in cardiac dysfunction [10]. Interestingly, despite the ubiquitous role of nuclear pore proteins, variants of *Nup93* appear to give tissue specific phenotypes.

This case demonstrates a novel phenotype of extrarenal manifestations that were not previously described in individuals with *NUP93* mutation. Unlike prior reported cases of *NUP93* mutations, our patient had neurological and cardiac involvement including significant developmental delay and cardiomyopathy.

Our patient’s genetic testing was negative for genes that cause Galloway-Mowat syndrome (GAMOS) though the constellation of glomerulopathy, central nervous system involvement might suggest this syndrome which was reported in other nucleoporin mutations [10–12].

When compared to idiopathic SRNS, genetic causes of SRNS usually do not recur after transplant hence these patients do not require higher immunosuppression as in idiopathic forms of SRNS [3, 6]. In a case series of four patients of Czech and Slovak patients with SRNS, one patient had recurrence of nephrotic syndrome 20 months after transplantation [4].

We describe novel mutations in the *NUP93* gene resulting in a syndromic phenotype with neurologic and cardiac features. It remains unclear if the *NUP93* variant contributed to her cardiomyopathy, as this gene has also been described as localizing to cilia as well as the nucleoporin. WES is the ideal test for patients with SRNS as a conclusive molecular diagnosis does influence therapeutic choices.

## Data Availability

Data sharing is not applicable to this article as no datasets were generated or analysed during the current study.

## Abbreviations

BMP7: Bone morphogenetic protein 7
ESRD: End-stage renal disease
FSGS: Focal segmental glomerulosclerosis
SRNS: Steroid-resistant nephrotic syndrome
WES: Whole exome sequencing
